# Integration of Physical Activity in Reablement for Community-Dwelling Older Adults: A Systematic Scoping Review

**DOI:** 10.1093/geroni/igab046.1613

**Published:** 2021-12-17

**Authors:** Hanne Leirbekk Mjøsund, Cathrine Moe, Elissa Burton, Lisbeth Uhrenfeldt

**Affiliations:** 1 Nord University, Bodø, Nordland, Norway; 2 Curtin University, Perth, Western Australia, Australia; 3 Nord University, Nord University, Bodø, Nordland, Norway

## Abstract

The aim of this study was to map evidence of how physical activity (PA) strategies are integrated and explored in research of interdisciplinary, time-limited reablement for community dwelling older adults and to identify knowledge gaps. Following an apriori protocol, we searched eight databases for eligible studies, in addition to citation and reference searches. Study selection and data-extraction was made independently by two reviewers. Fifty-one studies were included, showing that exercises and practice of daily activities were included in the majority of intervention studies, but in most cases little information about exercise components or strategies for increasing PA were provided. There was insufficient evidence for any synthesis of how reablement affects older adults’ PA levels, their physical fitness or how PA is experienced in reablement. There is a need to further investigate how the promotion of PA can be adequately implemented in reablement and how it may affect older adults’ function.

